# Axin2-expressing cells execute regeneration after skeletal injury

**DOI:** 10.1038/srep36524

**Published:** 2016-11-17

**Authors:** R. C. Ransom, D. J. Hunter, S. Hyman, G. Singh, S. C. Ransom, E. Z. Shen, K. C. Perez, M. Gillette, J. Li, B. Liu, J. B. Brunski, J. A. Helms

**Affiliations:** 1 Hagey Laboratory for Pediatric Regenerative Medicine, Division of Plastic and Reconstructive Surgery, Department of Surgery, Stanford University School of Medicine, Stanford, CA 94305-5148, USA; 2 Institute for Stem Cell Biology and Regenerative Medicine, Stanford University School of Medicine, Stanford, CA 94305, USA

## Abstract

The mammalian skeleton performs a diverse range of vital functions, requiring mechanisms of regeneration that restore functional skeletal cell populations after injury. We hypothesized that the Wnt pathway specifies distinct functional subsets of skeletal cell types, and that lineage tracing of Wnt-responding cells (WRCs) using the *Axin2* gene in mice identifies a population of long-lived skeletal cells on the periosteum of long bone. Ablation of these WRCs disrupts healing after injury, and three-dimensional finite element modeling of the regenerate delineates their essential role in functional bone regeneration. These progenitor cells in the periosteum are activated upon injury and give rise to both cartilage and bone. Indeed, our findings suggest that WRCs may serve as a therapeutic target in the setting of impaired skeletal regeneration.

Whether in health or disease, the skeleton must provide a range of functions, which depend upon mechanisms for repopulating functional cell types. Aside from classical functions in structural support and movement^1^, the skeleton acts as a supportive niche for hematopoiesis^2^,^3^,^4^, and an active regulator of endocrine homeostasis^5^,^6^. The coordination of regeneration is of particular interest, because mechanisms must be executed rapidly^7^. We sought to explore the intrinsic differences between cells that make up the skeleton; specifically, how different states of these cells collectively give rise to the regenerative response.

The development and maintenance of the adult mammalian skeleton have been thoroughly explored at the single cell level^8^,^9^. While cell populations contributing to the injury response may appear morphologically homogeneous, phenotypically and functionally distinct cell types have been identified taking part in bone regeneration. Transcriptional signatures of as-of-yet unidentified cells suggest that one or more subpopulations demonstrate enhanced regenerative potential^10^. As a result, the molecular regulation and specification of injury-responsive cell populations is a fundamental question in skeletal biology.

We have previously demonstrated the role of Wnt signaling in its control of multiple aspects of skeletal development and regeneration^11^,^12^,^13^,^14^,^15^,^16^,^17^,^18^. Wnt proteins function as short-range signals to maintain stem cells in multiple adult mammalian tissues^19^. Wnt proteins signal primarily through the intracellular protein β-catenin to activate transcription^20^. A universal transcriptional target of β-catenin-dependent Wnt signaling is Axin2, and its expression provides a reliable readout of cells responding to Wnt^21^,^22^. Genetic fate mapping of Axin2^+^ cells has identified stem and progenitor cells in several adult mammalian tissues^23^,^24^,^25^,^26^.

Wnt-responding cells (WRCs) have been viewed as undergoing a transient step of cell differentiation induced by local Wnt stimuli^20^. However, recent findings demonstrate that WRCs are a predetermined subset in the fetal stage, which are later responsible for skeletal maintenance throughout the life of the organism^18^. We hypothesize that such a program persists in adult-stage skeletal tissues to specify injury-responsive cell subsets. Herein, genetic lineage tracing of Axin2^+^ cells and their progeny identifies a unique population of injury-activated progenitor cells in the adult periosteum of long bone. The behavior of these genetically labeled cells reveals a previously unrecognized role for the Wnt pathway to program adult mesenchymal cells as injury progenitors that quickly enact a new regenerate after injury.

## Results

**Figure 1:**
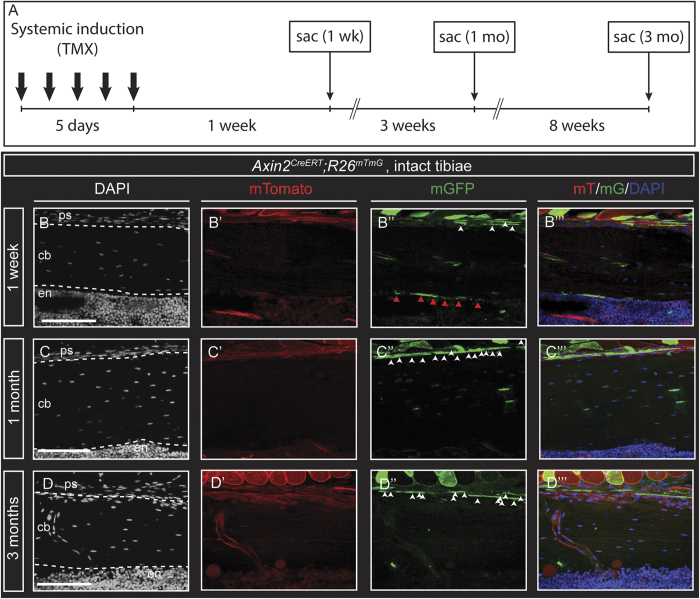
*Axin2*^*CreER*^;*R26*^*mTmG*^ labels a subset of skeletal cells in postnatal long bone that trace over the long term. (**A**), Schematic of experimental design. (**B-B”’**) Sagittal sections of *Axin2*^*CreER*^;*R26*^*mTmG*^ transgenic mice tibiae 1 week after 5 consecutive days of tamoxifen administration. Labeled cells and their progeny are found in the periosteum (**B-B”’**), white full arrows) and endosteum (**B-B”’**), red full arrows) 1 week after the induction period. GFP-labeled cells exhibited periosteal fates by 1 month (**C-C”**) through 3 months (**D-D”’**); endosteal (red full arrows) and trabecular bone-lining cells were rarely observed. Scale bars are equal to 100 μm. ps, periosteum; cb, cortical bone; en, endosteum.

To mark and follow the fates of WRCs and their progeny in the postnatal (adult) skeleton, we used 8-week-old tamoxifen-inducible *Axin2*^*CreER*^;*R26*^*mTmG*^ male mice to label Axin2^+^ cells in the tibia. In these experiments, Axin2^+^ cells are labeled with membrane GFP after tamoxifen administration (Fig. 1A). The GFP label is permanent, allowing for fate mapping of initially labeled cells and their descendants. Bones from *Axin2*^*LacZ*^ transgenic mice^27^,^28^, which express LacZ protein under the control of the endogenous *Axin2* promoter/enhancer region, showed expression in single cells within the endosteum, marrow stroma, and periosteum (Figure S1A,B). Bones from *Axin2*^*CreER*^;*R26*^*mTmG*^ (double-heterozygote) offspring^29^ showed GFP expression in single cells of each of these regions at 1 week after tamoxifen injection (Fig. 1B-B”’, quantified in Figure S1C,D), similar to the pattern observed in *Axin2*^*LacZ*^ transgenic mice. We have previously demonstrated that the skeletal phenotype of heterozygous *Axin2*^*LacZ*^ mice is identical to their wild-type littermates^30^. Within 1 week of initiating the trace, we observed GFP^+^ cells lining the periosteal (37.65 +/−1.28% of total cells; Figure S1C) and endosteal (42.16 +/−3.37% of total cells; Figure S1D) surfaces of cortical and trabecular bone. GFP^+^ cells expressed both Runx2 and Sox9, which are known mesenchymal transcription factors (data not shown). By 1 month, the proportion of labeled GFP^+^ cells increased substantially in the periosteum (61.89 +/−1.35% of total cells; Figure S1C), while that of the endosteum (2.38 +/−1.2% of total cells; Figure S1D) and marrow cavity had declined (Fig. 1C-C”’). By 3 months, GFP was widely expressed in the periosteal surfaces (65.54 +/−1.11%; Figure S1C) and within cortical bone (Fig. 1D-D”’), while labeled cells in the endosteum were rarely observed (0.64 +/−0.53%; Figure S1D). Of note, the total number of cells within the periosteum remained constant over the time points studied. Together these findings suggest that the periosteum is maintained over time, presumably by the proliferation of *Axin2* expressing progenitor cells.

**Figure 2:**
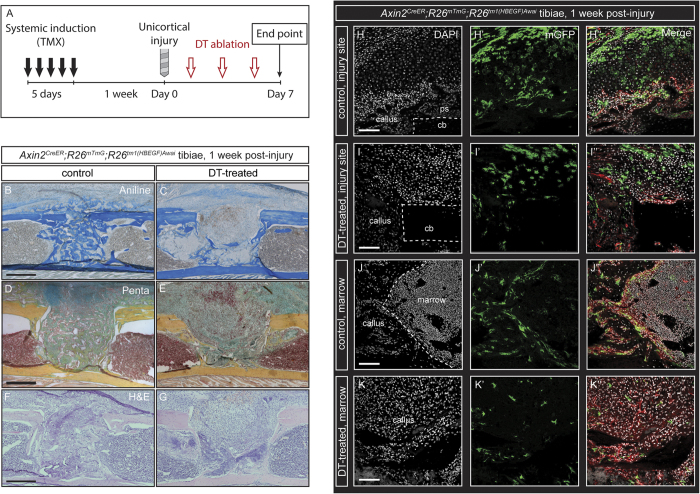
DTR-based ablation of Wnt-Responsive Cells (WRCs) disrupts bone regeneration and repair after injury. (**A**), Schematic of experimental design. (**A–B**) Aniline blue staining of sagittal sections of *Axin2*^*CreER*^;*R26*^*mTmG*^;*R26*^*tm1(HBEGF*)*Awai*^transgenic mice. PBS control (**B**) and DT-treated (**C**) tibiae showing reduced bone formation in DT-treated (decreased dark blue staining of collagen) tibiae as compared with control (increased dark blue staining of collagen) tibiae. The cellular morphology, proportional density, and organization of GFP^+^ cells differed markedly between DT-treated and control tibiae at 1 week post-injury. Movat’s pentachrome staining of control (**D**) and DT-treated (**E**) tibiae reveal higher levels of extracellular matrix (yellow to blue/green centrally) in controls versus increased cellular infiltrate (red centrally). Hematoxylin and eosin staining in representative control (**F**) and DT-treated (**G**) tibiae at 1 week after injury confirm differences in cellular density within the injury site. Histologic analysis of GFP and RFP fluorescence in control (**H-H”**) superficial callus, (**J-J”**) deep callus) and DT-treated (**I-I”**) superficial callus, (**K-K”**) deep callus) tibiae. GFP and DAPI are presented as individual channels and merged with RFP. Scale bars are equal to 100 μm. Dotted white lines outline injured cortical bone (**H,I**) and the deeper bony callus (**J**). ps, periosteum; cb, cortical bone.

Recent studies have shown that the neonatal skeleton contains Wnt-responsive osteoprogenitors that give rise to the postnatal skeleton and demonstrate sustained *Axin2*-expression, owing to autocrine stimulation via Wnt ligand secretion^18^. Given the role of WRCs as a developmental lineage and sustained Wnt ligand source, we probed the adult skeleton for Wnt-responding progenitor cells and investigated their response to injury. We tested the contribution of WRCs and their progeny to bone repair and whether ablation of the labeled cells could lead to a disruption in the rate of repair after injury. We crossed *R26*^*tm1(HBEGF*)*Awai*^mice, which express simian diphtheria toxin receptor (DTR) in a CreER-dependent manner, with *Axin2*^*CreER*^;*R26*^*mTmG*^ mice. 8-week-old triple heterozygous offspring (*Axin2*^*CreER*^;*R26*^*mTmG*^*; R26*^*tm1(HBEGF*)*Awai*^) were pulsed with tamoxifen, initiating expression of both GFP and DTR in WRCs only. 1 week after initiating the trace, these mice were skeletally injured and treated with either 100-ng diphtheria toxin (DT) in phosphate-buffered saline (PBS) or PBS alone (control). Sagittal sections from heterozygous *Axin2*^*CreER*^;*R26*^*mTmG*^ mice (n = 8) were compared to triple heterozygous *Axin2*^*CreER*^;*R26*^*mTmG*^*;R26*^*tm1(HBEGF*)*Awai*^ mice (*n* = 6) for GFP expression and histologic analysis and found to be equivalent in both injured and uninjured conditions (Fig. 2D, S2B).

We performed 1.0-mm unicortical drill defects in the tibiae to achieve a model of bone healing which recapitulates a fracture injury environment with minimal variation between animals. After injury, a soft tissue callus forms over the injury site within 1 week and ossifies after 2 weeks (Figure S2B,C). Histologic analysis using aniline blue staining for collagen revealed that bone regeneration in DT-treated mice was greatly reduced at 1 week compared to controls (Fig. 2B,C). In all 7 control calluses, GFP^+^ cells were found along endosteal surfaces and within bone at 1 week post-injury (Fig. 2H-H””). Additional controls were traced to 1 month (*n* = 4) and 3 months (*n* = 4) post-injury. GFP labeled cells were consistently observed along the periosteum and as osteocytes embedded in cortical bone over the 3-month post-injury interval (Figure S2F”’-G, I”’-J), similar to the cells that were initially labeled. The cellular morphology, proportional density, and organization of GFP^+^ cells differed markedly between DT-treated and control tibiae at 1-week post-injury (Fig. 2H–K”). Movat’s modified pentachrome staining revealed greater cellularity and reduced collagen deposition, evidenced by a higher ratio of red to blue/yellow staining, in treated as compared with control tibiae (Fig. 2D,E). This observation was confirmed via hematoxylin and eosin staining (Fig. 2F,G). Periosteal chondrogenesis was observed in both DT-treated and control samples (Fig. 2D,E) with relatively less ossification observed in DT-treated samples (Fig. 2B,C). Sites of chondrogenesis were observed in both deep and superficial callus regions of DT-treated samples that were not observed in control samples (Fig. 2H–K”), while control DT-injections in *Axin2*^*CreER*^;*R26*^*mTmG*^ mice which were not previously induced with tamoxifen (*n* = 3) had no observable effect on healing.

**Figure 3:**
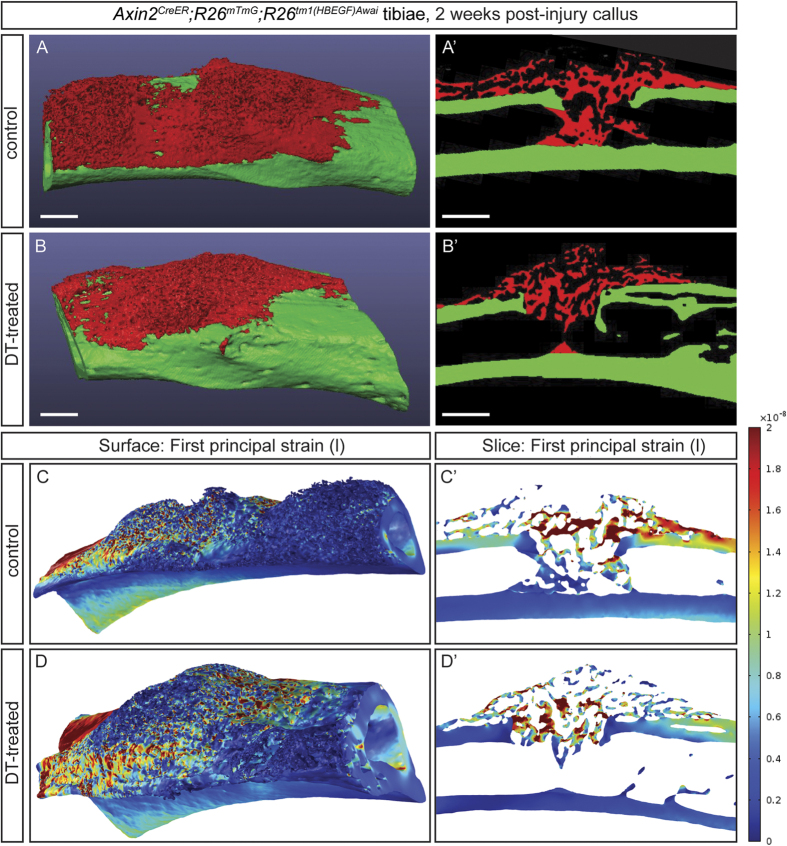
WRCs are essential for normal bone callus architecture and fracture stabilization after injury. (**A-B**’), μCT three-dimensional isosurface (**A,B**) and serial sections (**A’,B’**) from representative PBS-treated (**A-A**’) and DT-treated (**B-B’**) tibiae in *Axin2*^*CreER*^;*R26*^*mTmG*^;*R26*^*tm1(HBEGF*)*Awai*^transgenic mice delineate the woven bony regenerate (red, **A-B’**) versus the surrounding cortical bone (green, **A-B’**). Three-dimensional isosurface rendering and serial sections with finite element modeling of first principle (tensile) strain fields in representative PBS-treated (**C-C’**) and DT-treated (**D-D’**) tibiae in *Axin2*^*CreER*^;*R26*^*mTmG*^;*R26*^*tm1(HBEGF*)*Awai*^transgenic mice. The rainbow legend indicates tensile strain values within both isosurface and serial renderings. Scale bars are equal to 100 μm.

After 2 weeks post-injury, bone architecture within the injury site, measured via micro-computed tomography (μCT), were observed to be different in DT-treated as compared with PBS-treated controls (Fig. 3A,B’). According to Frost’s mechanostat theory, a refinement of Wolff’s law, a range of stresses and strains exists that correlates with increased bone mineral deposition^31^. Therefore, we further examined these ultrastructural and histological differences by employing two successive iterations of finite element (FE) modeling. While mechanical strength testing enables the functional assessment of biologic tissue, this modeling approach, whereby μCT data was integrated into the FE model, allowed us to specifically analyze the woven bony regenerate *in situ*, as opposed to the surrounding cortical bone. First, a simple two-dimensional model was created to highlight gross differences in the stresses and strains around the defect site for the control and experimental groups (Figure S3B–D). Control tibiae more effectively transferred the load across the defect site, while DT-treated tibiae more effectively dissipated the load along the remaining bony cortex. This was attributed to the softer material properties of the defect site in DT-treated tibiae compared to controls^32^,^33^,^34^,^35^. Next, a more complex three-dimensional model was generated to further explore these differences. In an effort to assess the functional response of the bony callus under loading, we simulated cantilever beam bending of the tibia. Finite element analysis of the first principal strain revealed more evenly distributed strain fields across the bony callus in the control tibiae in comparison to the DT-treated tibiae (Fig. 3C’), indicating that physiologic loads were more effectively transferred across the control tibiae in comparison to DT-treated tibiae. The average tensile, compressive, and total strains across the bony callus were lower in the control than the DT treated tibiae (9.14, 5.13, 7.13 nanostrain in control, respectively; versus 9.61, 5.22, 7.42 in the DT-treated, respectively). However, these differences are too small to draw conclusions about the functional difference between tibiae. Strain energy density was also calculated, acting as a predictor for mechanical fatigue failure. Higher stored strain energy densities correlates with a greater likelihood of failure^36^. The total strain energy density stored in the control sample was drastically lower than the DT-treated specimen (3.68E-6 J vs. 2.22E3 J). Taken together, these data confirm an important functional role for WRCs, both in terms of bony bridging and collagen deposition during bone repair.

**Figure 4:**
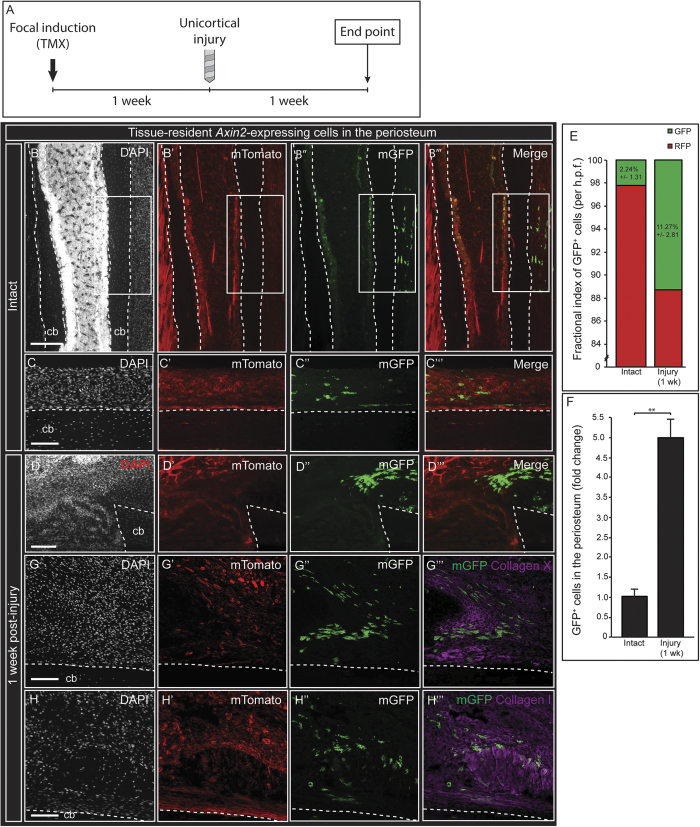
Injury activates tissue-resident WRCs that contribute to the healing callus in a manner that is spatially-restricted to the periosteum. (**A**), Schematic of experimental design. Focal induction of Axin2-expressing cells in the periosteum was performed by administering a single dose of 4-hydroxytamoxifen to the surface of the periosteum. (**B-C‘”**) 1 week after induction, GFP-labeled cells were observed and confined to the periosteum. GFP-labeled cells were not found in the marrow cavity or endosteum. 1 week after long bone injury, a proportional increase of GFP-labeled cells was observed in the callus (**D-D”’**, quantified in **E,F**). GFP-labeled cells were found in higher frequency within the periosteal injury callus. GFP-labeled cells of the periosteal callus colocalized with collagen type X (**G-G”’**), a marker of hypertrophic chondrocytes; and collagen type I (**H-H”’**), a matrix component of bone. Labeled cells were not observed contributing to the deeper bony callus of the marrow cavity, as demonstrated after systemic induction methods. cb, cortical bone. White boxes in (**B-B”’**) indicate the region of 4-hydroxytamoxifen application. Scale bars are equal to 100 μm. Dotted white lines outline the cortical bone before (**B-C”’**) and after injury (**D-D”’**), (**G-H”’**). cb, cortical bone.

The capacity of the periosteum to regenerate bone after perturbation has long been observed^37^,^38^. The periosteum has also been identified as a niche for progenitor cells that participate in endochondral ossification during postnatal fracture healing^39^. However, a challenge met by previous studies was the inability to maintain the physical identity of cells from the periosteum during the healing process, and effectively identify the periosteum as the cellular source after healing. This has prevented the accurate assessment of the contribution of skeletal cell compartments to fracture healing *in vivo*. Our consistent observation of labeled Axin2^+^ cells in the intact periosteum and the dynamic injury response of labeled cells in the periosteal callus prompted us to assess the contribution of tissue-resident periosteal cells to healing after fracture. To test this hypothesis, we developed a local induction method to label resident periosteal cells using 4-hydroxytamoxifen, allowing us to trace Axin2^+^ cells derived from the periosteum. One week after focal tamoxifen induction, GFP^+^ cells were observed in the periosteum, with infrequent labeling of surrounding soft tissues and deeper osteocytes. Importantly, labeled GFP^+^ cells were not observed in the marrow cavity or along the inner endosteum (*n* = 7; Fig. 4B-C”’, S4B-D”). 1 week after injury, GFP^+^ cells were observed in aggregates with organized polarity and similar phenotypes surrounding the defect site. In addition, we observed an increase in the proportion of GFP^+^ cells in the region of skeletal injury, supporting a cellular model of healing where GFP^+^ cells expand toward the defect in response to injury (Fig. 4D-D”’). In the periosteal callus, variation in cellular morphology and differentiation potential was observed. GFP^+^ cells colocalized with collagen type X (Fig. 4G-G”’), a marker of hypertrophic chondrocytes, and collagen type I, a marker of bone (Fig. 4H-H”’).This suggests that Axin2^+^ cells originating from the periosteum have the capacity to form both cartilage and bone, presumably through endochondral ossification. Conversely, the deeper callus of the marrow cavity primarily ossifies through intramembranous ossification. Notably, GFP^+^ periosteal cells were not observed in any regions of the deeper bony callus or surrounding marrow cavity and were strictly observed in the overlying periosteal callus. These data confirm a fracture healing model whereby periosteum-resident Wnt-responding cells contribute specifically to bridging of the overlying bony callus.

**Figure 5:**
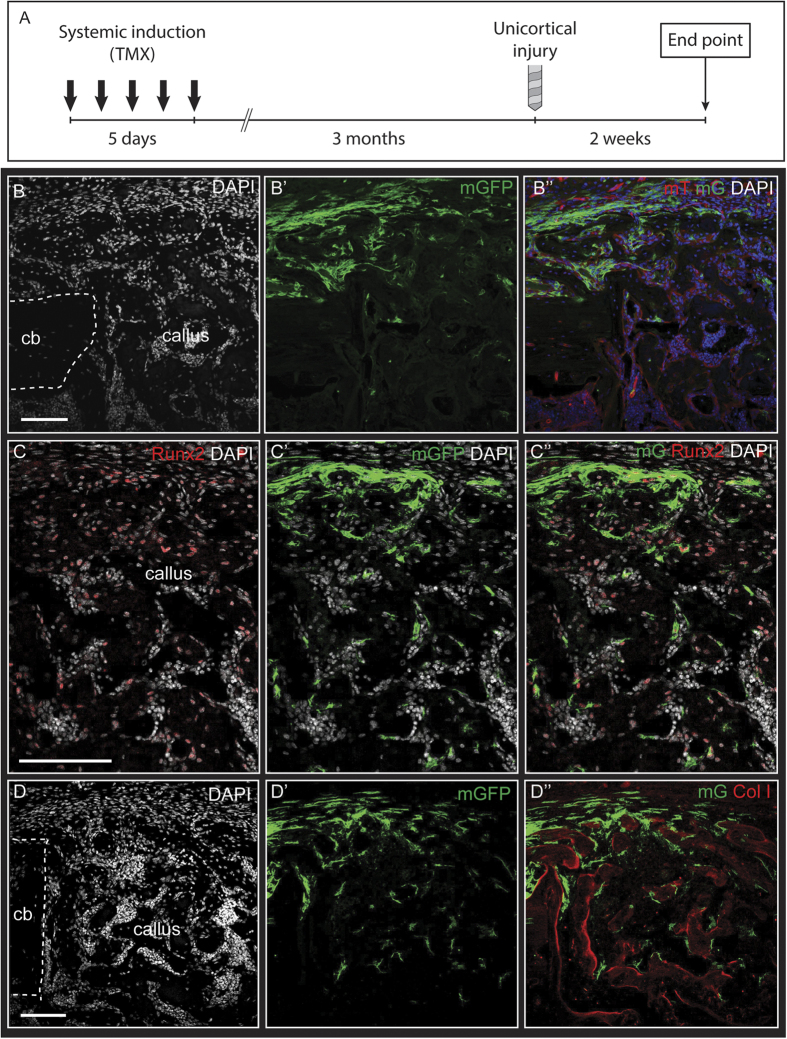
Long-lived WRCs in the periosteum are activated by skeletal injury. (**A**), Schematic of experimental design. 3 months after administration of tamoxifen, unicortical skeletal injuries were performed. Sagittal sections of injured tibia 2 weeks after injury revealed GFP-labeled cells confined to the periosteum and superficial bony callus (**B-B”**). GFP-labeled cells were observed expressing Runx2 (**C-C”**) and lining regions of newly-formed bone in the callus, as identified by collagen type I staining (**C-C”’**). Merged image in (**B”**) indicates merged mTomato, mGFP, and DAPI filters. Merged image in (**C”**) indicates merged mGFP, Runx2, and DAPI filters. Merged image in (**D”**) indicates merged mGFP, Collagen type I, and DAPI filters. Scale bars are equal to 100 μm. Dotted white lines indicate the injured bony cortex. cb, cortical bone.

To further explore the role of WRCs as progenitors of the adult skeleton, *Axin2*^*CreER*^;*R26*^*mTmG*^ mice were induced and GFP-labeled cells were traced over a 3-month period for additional analyses. These populations were observed to expand within the periosteum over the 3-month interval (Fig. 1D-D”’, S1C). The regenerative capacity of these cells was tested using an injury model 3 months after initial labeling. Tibiae were harvested at 2-week post-surgery. Confocal microscopy of a 3-month traced long bone at 2-weeks post-injury shows long-lived GFP^+^ cells present in the periosteal callus and largely absent in the bone marrow cavity (Fig. 5B-D”), aligning with tracing data in intact tissues (Fig. 1D-D”’) and data obtained using focal induction of periosteum tissue (Fig. 4B-D”’). These cells display robust formation of bone through endochondral ossification within the periosteum, and minimal contribution to the intramembranous bone formation in the marrow cavity. An increase in the proportion of GFP^+^ cells was observed in the superficial callus and periosteum compared to the deeper callus and surrounding marrow cavity. GFP^+^ cells colocalized with both Runx2 (Fig. 5C-C”) and collagen type I (Fig. 5D-D”) immunostaining, with increased abundance of both GFP^+^Col 1^+^ and GFP^+^Runx2^+^ cells in the periosteal callus as compared to the deeper injury callus. This suggests that the molecular phenotype of long-lived WRCs within intact periosteum is distinct from their daughter cells post-injury, which is consistent with a progenitor characteristic of bone. The absence of endosteal WRCs over the long term, and their lack of contribution to intramembranous bone formation, is suggestive of a skeletal population that is more differentiated than its periosteal counterparts. Altogether, these data reveal that the adult skeleton contains WRCs that demonstrate conserved progenitor characteristics in a manner that is spatially restricted to the periosteum. The periosteum contains subsets of WRCs that display an enhanced injury response, give rise to fate-restricted cells, and retain the frequency of WRCs. These cells thus offer a therapeutic target to increase or restore the regenerative capacity of bone.

## Discussion

The molecular regulation of injury-responsive progenitor populations in the mammalian skeleton has remained elusive. Previous studies have characterized the skeletal lineage by phenotyping surface molecule expression of functionally distinct populations and comparing their unique RNA signatures. Others have similarly described a skeletal progenitor by prospectively fate mapping the cells responding to the bone morphogenetic protein (BMP) pathway. These studies demonstrated the existence of skeletal stem and progenitor cells that could be traced from development. Moreover, recent data in neonates demonstrates that Axin2^+^ cells are a predetermined subset of cells that give rise to the postnatal skeleton. Our study enhances this model by lineage tracing Axin2-expressing cells in the adult skeleton and characterizing their molecular phenotype and contribution to healing after injury.

### Injury activation of WRCs leads to migration and subsequent endochondral ossification

Adult stem cells are maintained in a quiescent state but are able to rapidly expand and differentiate in response to stress or insult. Under normal turnover, Axin2^+^ progenitor cells were labeled over the long-term, maintaining the periosteum. This progenitor characteristic is further supported by their expansion in response to skeletal injury. The expansion in WRCs observed after injury may be driven by multiple processes: proliferation, migration of circulating cells, or local migration of resident cells. Focal induction of tissue resident WRCs demonstrate a parallel expansion after injury. The absence of labeled cells in circulation during the focal induction assay supports the tissue-resident characteristic of these cells. However, proliferation and cell migration are not mutually exclusive over the time course of healing and, therefore, cannot be ruled out. Taken together, our findings reveal a model for Wnt pathway specification of injury-responsive progenitors in the adult skeleton and may serve as a therapeutic target to enhance or restore the regenerative capacity of bone. Future efforts to identify the gene-expression patterns of WRCs may reflect observed phenotypic changes at the transcriptome level.

### Implications in translational medicine: Regeneration and Cancer

Our study of WRCs imply a therapeutic potential of this population to treat osteoarthritis, osteoporosis, poor-healing diabetic fractures, and critical-sized segmental bone defects. Further research toward the *in vivo* directed differentiation of WRCs may represent a paradigm shift in tissue engineering to produce cartilage and/or bone. WRC engraftment into aged or diseased skeletal tissue may also rejuvenate and heal previously untreatable conditions. Importantly, recent studies demonstrate that aberrant activation of Wnt signaling drives unregulated proliferation in osteosarcoma cells. Development of osteosarcomas is driven by cancerous skeletal cells with enhanced osteogenic properties; while downregulation of Wnt signaling with pathway inhibitors, such as dnTCF4 or siLEF1, induces cell cycle arrest and suppresses osteosarcoma growth^34^. Indeed, these cells may serve as a therapeutic target for treatment of osteosarcoma, the most prevalent tumor of bone in the pediatric population. Therefore, our findings in basic science have interesting translational implications for therapeutically targeting injury-responsive skeletal stem and progenitor cells across multiple patient populations.

## Methods

### Mice

Mice were bred and maintained at the Stanford University Research Animal Facility in accordance with Stanford University guidelines. All experimental protocols followed ARRIVE (Animal Research Reporting of *In Vivo* Experiments) guidelines and were approved by the Stanford University Administrative Panel on Laboratory Animal Care. All the animals were housed in sterile micro-insulators and given water and rodent chow ad libitum. *Axin2*^*CreER*^ and *Axin2*^*LacZ*^ strains were obtained from Jackson laboratories. The *ROSA26*^*mTmG*^ (*R26*^*mTmG*^) reporter mice, which harbor a double-fluorescent reporter that permanently replaces the expression of membrane-bound tomato red fluorescent protein (RFP) with membrane-bound GFP after recombination^29^, and the *R26*^*tm1(HBEGF*)*Awai*^ mice were obtained from Jackson laboratories. *Axin2*^*CreER*^ transgenic mice were crossed with *R26*^*mTmG*^ reporter mice and offspring were crossed with *R26*^*tm1(HBEGF*)*Awai*^mice. *Axin2*^*CreER*^; *R26*^*mTmG*^ offspring were used to trace *Axin2*-lineage–positive cells, defined *in vivo* by their GFP positivity, within long bones. Tamoxifen (Sigma-Aldrich) was dissolved in 90% corn oil/10% (vol/vol) ethanol, and filtered through a 0.2 μm membrane. 8-week old mice were administered intraperitoneal injections with tamoxifen at 200 mg/kg daily for five consecutive days, except where indicated otherwise. Unicortical drill defects were performed 1 week after the final administration of tamoxifen.

### Focal induction for periosteum labeling studies

8-week old mice were placed under 2% (vol/vol) isoflurane anesthesia, and after administration of subcutaneous buprenorphine (0.1 mg/kg) the mouse was placed in the supine position. A longitudinal incision was made in the skin parallel to the long-bone, subcutaneous fat and muscles were divided parallel to muscle fibers. 10 μg of tamoxifen was applied to the surface of the periosteum using a 2.0 μL Hamilton Modified Microliter Syringe (Sigma-Aldrich) at a recorded distance from the tibial tuberosity, taking care not to damage the periosteum. Skin was subsequently closed and the mice monitored postoperatively according to protocol. After a 1-week period, mice received skeletal injuries as specified. Results of focal induction assays were tested and confirmed in a second set of independent experiments.

### Unicortical drill injury model

Unicortical drill defects were performed as previously described^30^. Briefly, 8-week old mice were placed under 2% (vol/vol) isoflurane anesthesia, and after administration of subcutaneous buprenorphine (0.1 mg/kg) the mouse was placed in the supine position. A longitudinal incision was made in the skin parallel to the long-bone, subcutaneous fat and muscles were divided parallel to muscle fibers, and a single 1-mm Unicortical defect was made taking care not to damage the surrounding soft tissues and periosteum. Skin was subsequently closed and the mice monitored postoperatively according to protocol.

### Sample processing and histology

Tibiae collected were immediately fixed in 4% paraformaldehyde (wt/vol) overnight at 4 °C. The next day, they were decalcified in 19% EDTA, pH 7.4 [10% tetrasodium salt, 9% disodium salt (wt/vol] for 21 d at 4 °C, changing every day. The tibiae were incubated in 30% (wt/vol) sucrose at 4 °C overnight, then embedded in OCT. For histology, H&E, pentachrome, and aniline blue staining was performed on cryosectioned slides, further dehydrated up to 100% ethanol, and mounted with Permount.

### Finite element modeling

Finite element modeling was generated in COMSOL Multiphysics (version 5.1; COMSOL Inc., Burlington, MA, USA). The model was comprised of two iterations. First, two-dimensional modeling was created from the geometry of histological sections for the control and experimental groups at 1 week post-injury. Primitive shapes were used to estimate the relevant geometries. Linear elastic material properties were assigned to cortical bone, marrow, bone callus, and fibrocartilage, found in Table S1. The deeper cortex opposite to the drill injury was subjected to a normally applied upward load of 1 N/m inducing bending. Subsequent three-dimensional modeling was obtained from fixed murine tibiae from control and experimental groups. The morphology of the bone was scanned from a dry sample using μCT (Micro XCT, Xradia Inc, Pleasanton, CA) at 4× magnification, 10 μm resolution. The morphological data were reconstructed in a three-dimensional solid volume in the 3D image data visualization program ScanIP (Version 7.0, Simpleware Ltd). Following the segmentation process to isolate separate cortical bone and bony callus, a mesh was generated using free tetrahedrals consisting of 3.5E6 elements (average element quality.56) per sample. The mesh was exported as an.mph file for analysis in COMSOL Multiphysics. All model components were assumed to be isotropic homogeneous linear elastic materials. Material properties used are shown in Table S1. The proximal boundary of each tibia was fixed and distal boundary loaded with 10 N/m to simulate cantilever beam bending. This was done to simulate posterior bending, a physiologic loading regimen.

We noticed that although some differences were apparent, the average, min, and max for stress and strain between the control and DT-treated groups were similar. However, upon evaluation of the stored strain energy density of the two samples, we found pronounced differences. Deformed materials store energy similar to a coiled spring. The local concentration of this energy is the scalar strain energy density (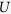). In members with higher stresses, the strain energy density is higher. Evaluating strain energy density is a predictor of fatigue failure^36^. Since a member that is more highly stressed will have a higher strain energy density, it requires less external effort to reach failure.


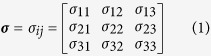



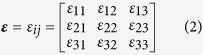


where σ is the Cauchy stress tensor and ε is the Cauchy strain tensor of a material. Our model employed an isotropic assumption for each material. The derivation of the isotropic strain energy density function follows as:


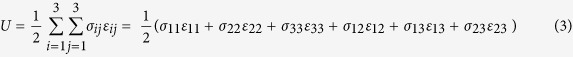


Between the two specimens, the total strain energy density was dramatically different (3.68E-6 vs. 2.216E3 J for control vs. experimental).

### Immunostaining

Frozen samples were sectioned at 10 μm using CryoJane (Leica Microsystems). The slides were blocked in 1× Power Block (BioGenex) in PBS at room temperature for 1 h, then stained with primary antibody diluted in blocking buffer overnight at 4 °C. The next day, the slides were washed in PBS 3 × 5 min, incubated in secondary antibody in blocking buffer for 1 h at room temperature, and washed in PBS 3 × 5 min before mounting with DAPI (Vectashield). The primary antibodies used were goat anti-GFP (FITC) (1:200; abcam) and rabbit anti-beta galactosidase (abcam). Anti-Rabbit-AlexaFluor488 (Life Technologies) was used for the secondary antibody for immunofluorescent staining.

### Microscopy

Laser scanning confocal microscopy was performed using a Leica TCS SP8 × confocal microscope (Leica Microsystems) with an objective lens (10× HC PL APO, air, N.A. 0.40; 20× HC PL APO IMM CORR CS2, H2O/Glycerol/oil, N.A. 0.75), located in the Cell Sciences Imaging Facility (Stanford University, Stanford, CA).

### Fluorescence quantification

Randomly chosen sections (25 per sample) from all *Axin2*^*CreER*^ bones were analyzed using ImageJ software. Analysis was performed by counting GFP^+^ cells and total DAPI^+^ nuclei within an ROI assigned to the periosteum. The values calculated between sections were averaged across animal subjects for a given condition and presented as mean percentages. All data were quantified and ROIs assigned by a blinded observer from serial sections of confocal micrographs analyzed using ImageJ and Adobe Photoshop CS6 (Adobe Systems, San Jose, California).

### Statistics

All statistical analysis was performed in GraphPad Prism (GraphPad Software) using a Student’s *t*-test assuming two-tailed distribution and unequal variances. Data are expressed as mean ± standard deviation of the mean. *P* values ≤ 0.05 were considered statistically significant.

### DTR-based ablation of WRCs (GFP^+^) after tibia drill injury

*Axin2*^*CreER*^;*R26*^*mTmG*^;*R26*^*tm1(HBEGF*)*Awai*^ mice were injected (4×) intraperitoneally with either 100 ng DT in 100 ul PBS (*n* = 6) or 100 ul PBS alone (*n* = 6) at the time of drill injury (day 0), and every other day until mice were sacrificed and bones were harvested. Additional controls were also established by administering DT in a similar fashion to *Axin2*^*CreER*^;*R26*^*mTmG*^ mice (*n* = 3). Tibiae were allowed to heal in DT-treated (*n* = 6) and control mice (*n* = 6) until killing at post-operative day 7, at which point tibiae were harvested for histologic analysis of GFP/RFP fluorescence and connective tissue deposition by Aniline Blue and Movat’s Pentachrome staining.

## Additional Information

**How to cite this article**: Ransom, R. C. *et al.* Axin2-expressing cells execute regeneration after skeletal injury. *Sci. Rep.* **6**, 36524; doi: 10.1038/srep36524 (2016).

**Publisher’s note**: Springer Nature remains neutral with regard to jurisdictional claims in published maps and institutional affiliations.

## Supplementary Material

Supplementary Information
