# Supplementary material for: Axin2-expressing cells execute regeneration after skeletal injury
Source: Sci Rep. 2016 Nov 17;6:36524. doi: 10.1038/srep36524 (PMC5113299; doi:10.1038/srep36524)
Supplement: Supplementary Information [file srep36524-s1.pdf]

Axin2-expressing cells execute regeneration after skeletal injury

Ransom RC, Hunter DJ, Hyman S, Singh G, Ransom SC, Shen EZ, Perez KC, Gillette M, Li J, Liu B, Brunski JB, Helms JA

Supplemental Information

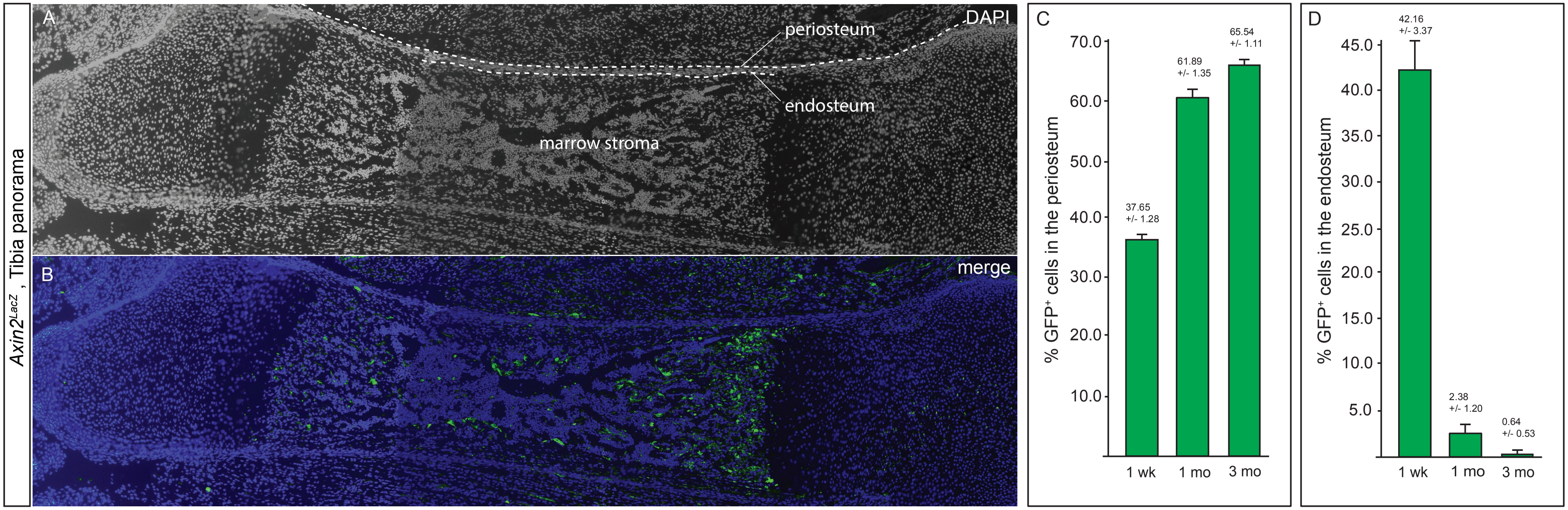

Figure S1 | a-b, Sagittal section through *Axin2^{LacZ}* transgenic 8 week old mouse tibia stained for DAPI (a) and beta-galactosidase (b). Quantification of GFP+ cells in the periosteum (c) and endosteum (d) over 3-month interval in *Axin2^{CreER};R26^{mTmG}* transgenic mice. Scale bars are equal to 100 micrometers.

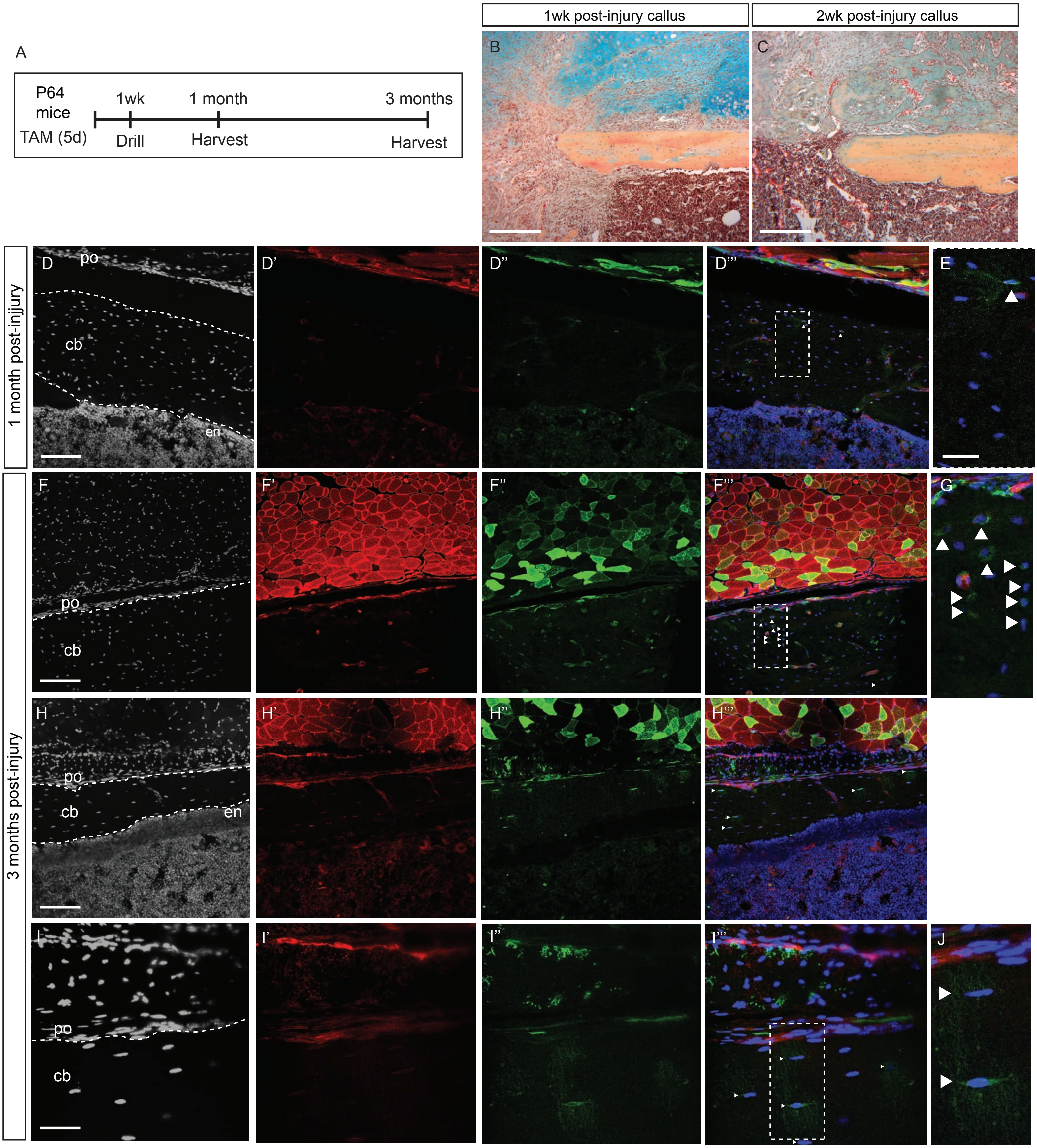

Figure S2 | a, Schematic of experimental design. Movat's pentachrome staining of tibiae at 1 week (b) and 2 weeks post-injury (c). Sagittal sections of *Axin2*^{CreER}*;R26*^{mTmG} transgenic mice tibiae at 1 month post-injury (d-e). Sagittal sections of *Axin2*^{CreER}*;R26*^{mTmG} transgenic mice tibiae at 3 months post-injury (f-j). po, periosteum; cb, cortical bone; en, endosteum. Scale bars are equal to 100 micrometers.

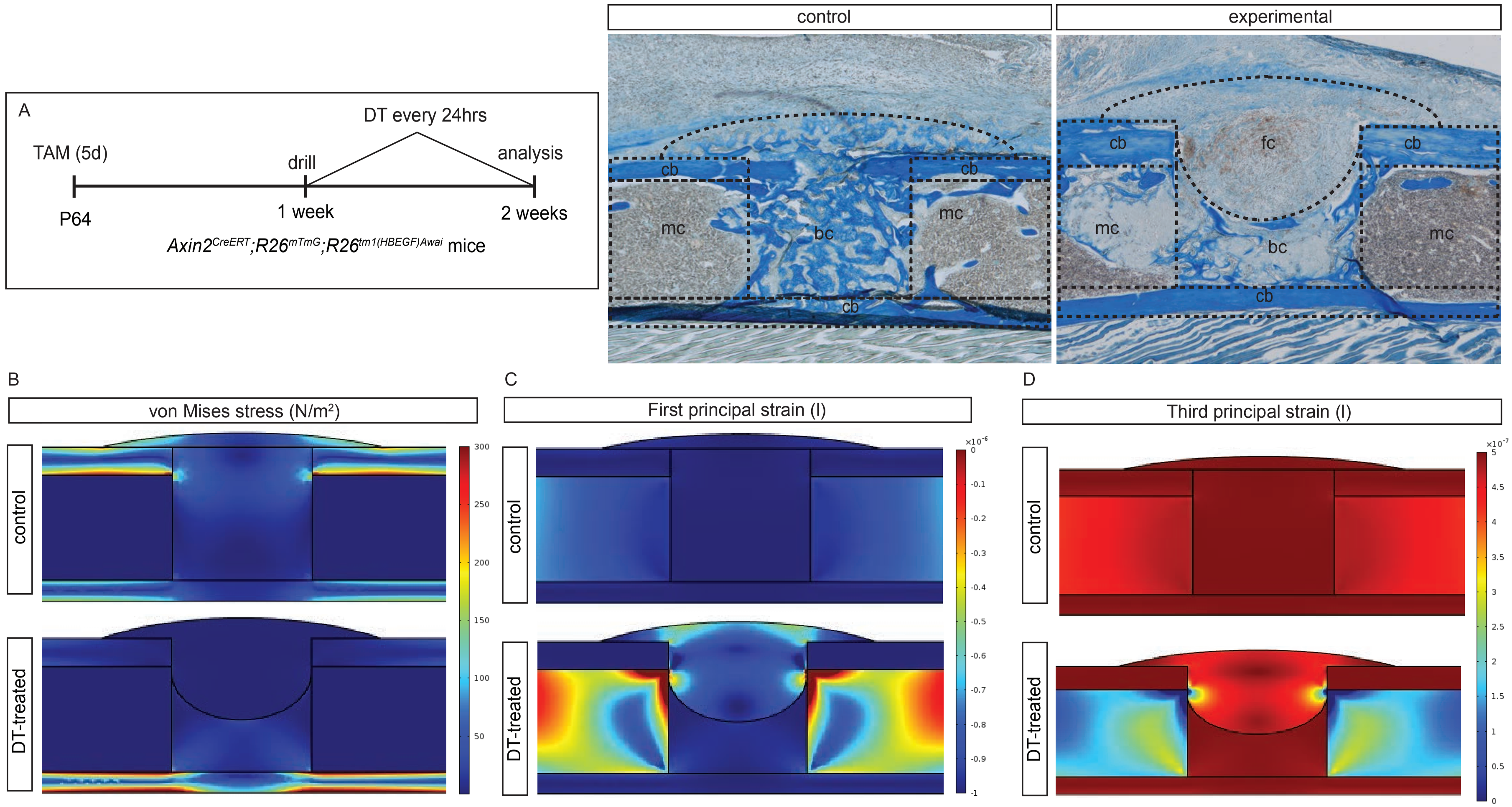

Figure S3 | a, Schematic of experimental design. Two-dimensional finite element modeling of stress (b), tensile strain (c), and compressive strain (d) across PBS-treated and DT-treated tibiae at 1 week post-injury. Scale bars are equal to 100 micrometers.

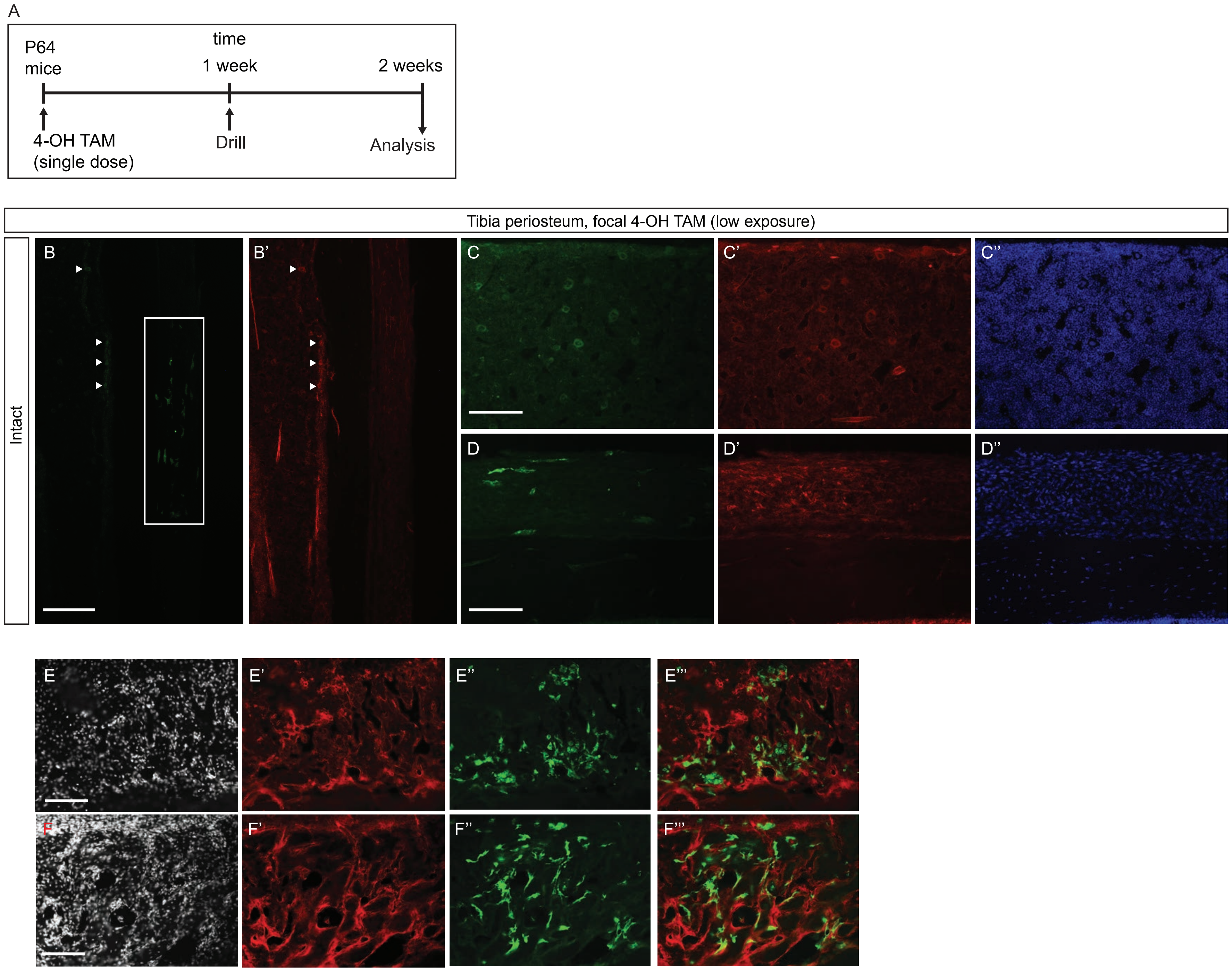

Figure S4 | a, Schematic of experimental design. Sagittal sections of intact tibiae 1 week after focal induction (b-d'') at low exposure, demonstrating GFP fluorescence in the periosteum (boxed in b, d-d'') with autofluorescent overlap in the endosteum (b-b') and marrow cavity (c-c''). White closed arrows indicate areas of autofluorescence. Sagittal sections of focally-induced injured tibiae at 1 week post injury (e-f'''). Scale bars are equal to 100 micrometers.

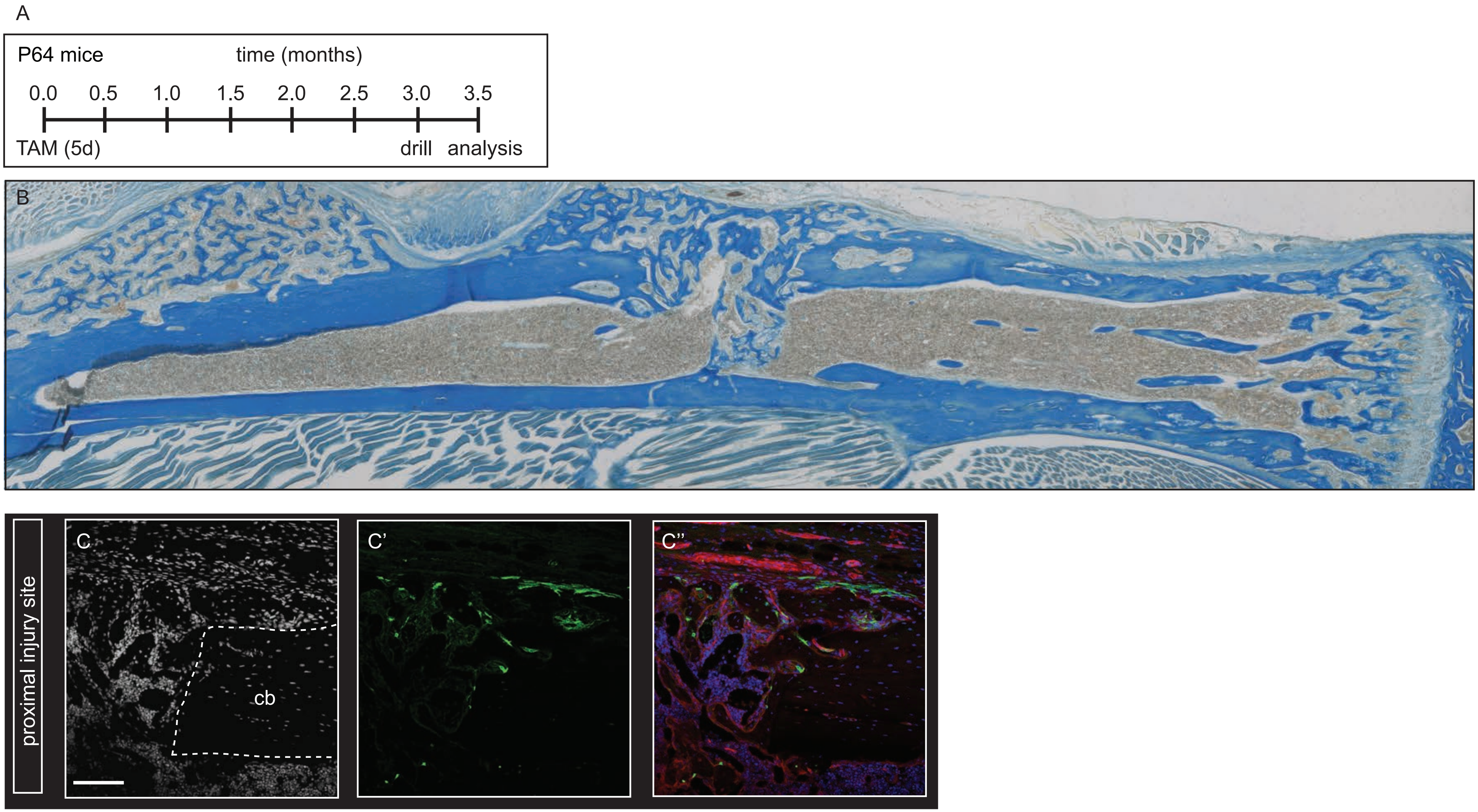

Figure S5 | a, Schematic of experimental design. Aniline blue staining of tibiae at 2 weeks post injury (b). High magnification of proximal injury site at 2 weeks after injury (c, from figure 5a). Scale bars are equal to 100 micrometers.

Table S1. Material properties of tissues after skeletal injury

Control	Elastic Modulus	Density (kg/m^3)	Poisson's Ratio
cortical bone	9 [GPa]	1700	0.3
deep callus	7.2 [GPa]	1560	0.35
superficial callus	5.85 [GPa]	1455	0.35
marrow cavity	30 [kPa]	900	0.49

DT-treated	Elastic Modulus	Density (kg/m^3)	Poisson's Ratio
cortical bone	9 [GPa]	1700	0.3
deep callus	0.9 [GPa]	1070	0.35
fibrocartilage	5 [GPa]	1200	0.35
marrow cavity	30 [kPa]	900	0.49

Table S1 | Material properties of tissues used for two-dimensional finite element analysis at 1 week after skeletal injury.
